# Distribution of Paraoxonase-1 (PON-1) and Lipoprotein Phospholipase A2 (Lp-PLA2) across Lipoprotein Subclasses in Subjects with Type 2 Diabetes

**DOI:** 10.1155/2018/1752940

**Published:** 2018-11-05

**Authors:** Angelina Passaro, Giovanni Battista Vigna, Arianna Romani, Juana M. Sanz, Carlotta Cavicchio, Gloria Bonaccorsi, Giuseppe Valacchi, Carlo Cervellati

**Affiliations:** ^1^Department of Medical Sciences, Internal Medicine and Cardiorespiratory Section, University of Ferrara, Via Aldo Moro 8, 44124 Ferrara, Italy; ^2^Medical Department, Internal Medicine Unit, Azienda Ospedaliera-Universitaria S. Anna, Via Aldo Moro 8, Ferrara, Italy; ^3^Department of Life Sciences and Biotechnology, University of Ferrara, Via Luigi Borsari 46, 44121 Ferrara, Italy; ^4^Department of Morphology, Surgery and Experimental Medicine, Section of Obstetrics and Gynecology, University of Ferrara, Via Aldo Moro 8, 44124 Ferrara, Italy; ^5^Menopause and Osteoporosis Centre, University of Ferrara, Via Boschetto 29, 44124 Ferrara, Italy; ^6^Plants for Human Health Institute, Animal Science Dept., NC Research Campus, North Carolina State University, 600 Laureate Way, Kannapolis, NC 28081, USA; ^7^Department of Biomedical and Specialist Surgical Sciences, Section of Medical Biochemistry, Molecular Biology and Genetics, University of Ferrara, Via Luigi Borsari 46, 44121 Ferrara, Italy

## Abstract

Paraoxonase-1 (PON1) and lipoprotein phospholipase A2 (Lp-PLA2) may exert an important protective role by preventing the oxidative transformation of high- and low-density lipoproteins (HDL and LDL, respectively). The activity of both enzymes is influenced by lipidome and proteome of the lipoprotein carriers. T2DM typically presents significant changes in the molecular composition of the lipoprotein subclasses. Thus, it becomes relevant to understand the interaction of PON1 and Lp-PLA2 with the subspecies of HDL, LDL, and other lipoproteins in T2DM. Serum levels of PON1-arylesterase and PON1-lactonase and Lp-PLA2 activities and lipoprotein subclasses were measured in 202 nondiabetic subjects (controls) and 92 T2DM outpatients. Arylesterase, but not lactonase or Lp-PLA2 activities, was inversely associated with TD2M after adjusting for potential confounding factors such as age, sex, smoking, body mass index, hypertension, and lipoprotein subclasses (odds ratio = 3.389, 95% confidence interval 1.069–14.756). Marked difference between controls and T2DM subjects emerged from the analyses of the associations of the three enzyme activities and lipoprotein subclasses. Arylesterase was independently related with large HDL-C and small intermediate-density lipoprotein cholesterol (IDL-C) in controls while, along with lactonase, it was related with small low-density lipoprotein cholesterol LDL-C, all IDL-C subspecies, and very low-density lipoprotein cholesterol (VLDL-C) in T2DM (*p* < 0.05 for all). Concerning Lp-PLA2, there were significant relationships with small LDL-C, large IDL-C, and VLDL-C only among T2DM subjects. Our study showed that T2DM subjects have lower levels of PON1-arylesterase compared to controls and that T2DM occurrence may coincide with a shift of PON1 and Lp-PLA2 towards the more proatherogenic lipoprotein subclasses. The possibility of a link between the two observed phenomena requires further investigations.

## 1. Introduction

Several lines of evidence clearly suggest that oxidative stress (OxS) is implicated in the pathogenesis of type 2 diabetes mellitus (T2DM) and plays a critical role in the development of its frequent microvascular and macrovascular complications [1]. OxS appears to mediate hyperglycemia-induced tissue damage by influencing polyol and the hexosamine pathway, increasing intracellular formation of advanced glycation end-products (AGEs) and their receptors (RAGEs) etc. [2]. OxS is both a downstream and upstream event of these altered processes. As paradigmatic example in this context, AGEs-RAGE interaction is accelerated by reactive oxygen species (ROS) and, in the same time, promotes the formation of these oxidants by inducing mitochondria dysfunction and nicotinamide adenine dinucleotide phosphate oxidase (NOX) activation [1, 2].

Increase in ROS results in the accumulation of oxidative-damaged biomolecules, including the highly proatherogenic oxidized low-density lipoproteins (ox-LDLs) [3, 4]. These modified lipoproteins entail endothelial cell activation, dysfunction, and death and contribute to the onset and progression of the atherosclerotic process [4]. This detrimental action of ox-LDL is antagonized by high-density lipoproteins (HDLs) which are able not only to promote reverse transport of cholesterol but also to act as effective anti-inflammatory and antioxidant agents [5, 6]. A wealth of *in vitro* and *in vivo* evidence suggests that paraoxonase 1 (PON1) and lipoprotein-associated phospholipase A2 (Lp-PLA2) contribute to vasculoprotective function of HDL [7–10]. Both enzymes are able to hydrolyze, by different and still poorly known mechanisms, lipo-lactones, such as those resulting from oxidation of fatty acid or cholesterol-enriching lipid environment of HDL and LDL [7, 9, 11]. It has been suggested that the antioxidant-like function of PON1 and Lp-PLA2 may account for the several findings linking altered levels of enzyme activities and the risk of developing T2DM as well as its related clinical complications [12–17].

HDL and LDL are heterogeneous collection of particles which vary in size, density, lipid composition, proteome, and physiological role [18]. The different biochemical dynamics of lipoprotein subclasses inevitably result in a different affinity between them and accessory proteins, such as Lp-PLA2 and PON1 [19–22]. A limited number of studies addressed the distribution of PON1 and Lp-PLA2 in HDL and LDL subclasses, respectively, and generated inconsistent results [6, 20, 21, 23].

In T2DM, the primary quantitative lipoprotein abnormalities are elevated triglyceride levels and diminished HDL-C levels; concomitantly, the lipoproteins also change in structure, chemical composition, and size [24]. In particular, the proportions of circulating small dense LDL and HDL are increased, while there are fewer particles of the respective large subclasses, leading to a more proatherogenic setting [24]. This qualitative change in lipoprotein might have significant repercussion in stability and activity of Lp-PLA2 and PON1; indeed, it is well known that both are sensitive to their milieu, intended as lipid and proteome that surround the two enzymes [25, 26]. Besides, OxS, high glucose levels, and inflammation have been repeatedly shown to induce modifications in the PON1 structure that, in turn, compromise its biological function [9, 11, 27, 28]. Overall, this convergent evidence makes it relevant to discern the interplay between PON1 and Lp-PLA2 with lipoprotein subclasses in the diabetic state.

To address this still open question, the present population-based study sought to determine the link of T2DM with PON1 and Lp-PLA2 activities and, most importantly, to evaluate whether the disease might affect the distribution of these two enzymes across lipoprotein subclasses.

## 2. Materials and Methods

### 2.1. Subjects

The subjects examined in this study were enrolled among men/women attending the metabolic outpatient clinic of Sant'Anna University Hospital (Ferrara, Italy) and outpatients undergoing bone densitometry testing at the Menopause and Osteoporosis Centre of the University of Ferrara [29]. Exclusion criteria for subjects with and without T2DM were infection, acute or chronic disease (affecting liver, kidney, lungs, etc.), dementia, cancer, pregnancy, and alcohol consumption > 10 g daily. The diagnosis of T2DM was made in agreement with American Diabetes Association (ADA) criteria. The whole number of participants was 719 and included 574 nondiabetic subjects (controls) and 145 T2DM subjects.

At the point of study entry, all participants underwent a clinical (questionnaire plus blood pressure), physical (weight, height, and waist circumference), and routine biochemical investigation (plasma lipid profile and glucose) by trained personnel. Standard questionnaire was administered to collect main demographic and clinical data (history of CVD and other complications of DM, smoking, and current medications). Participants were deemed hypertensive when having a mean systolic blood pressure ≥ 140 mmHg and/or mean diastolic blood pressure ≥ 90 mmHg and/or when they were on active antihypertensive treatment. Patients were defined as dyslipidemic, according to the National Cholesterol Education Program Adult Treatment Panel III 2004 [30] guidelines, when total cholesterol ≥ 200 mg/dL and/or LDL-C ≥ 130 mg/dL and/or HDL-C < 40 mg/dL and <50 mg/dL for males and females, respectively, and/or mean triglycerides ≥ 200 mg/dL and/or when on active lipid-lowering treatment (10 and 15% of controls and T2DM patients, respectively).

The whole study conforms to The Code of Ethics of the World Medical Association (Declaration of Helsinki) and was conducted accordingly to Good Clinical Practice guidelines. It was approved by the Local Ethics Committee; written informed consent was obtained from each patient and no personal information was available to the authors.

Of note, lipoprotein subclass analysis was carried out in a subsample of 292 subjects (202 controls and 90 T2DM patients) that will be referred with the term Lipoprint throughout the report.

### 2.2. Biochemical Assays

Venous blood samples from all study participants were drawn after overnight fasting, and serum was stored at −80°C until analysis.

Arylesterase and lactonase activities of PON1 and Lp-PLA_2_ activity in serum were measured by UV-VIS spectrophotometric assays in a 96-well plate format by using a Tecan Infinite M200 microplate reader (Tecan Group Ltd., Switzerland).

Arylesterase activity was measured by using phenylacetate as substrate [31]. A molar extinction coefficient (wavelength = 270 nm) of 1.3 × 103 L^−1^·mol^−1^·cm^−1^ was used for the calculation of enzymatic activity, which was expressed in kilo unit per liter. One unit of arylesterase activity accounts for 1 *μ*mol of phenol produced in a minute under the conditions of the assay. Intra-assay CV was 3.8% whereas interassay CV was 9.7% [31].

Lactonase activity was assessed using gamma-thiobutyrolactone (TBL) as substrate, and Ellman's procedure was used to spectrophotometrically monitor the accumulation of free sulfhydryl groups via coupling with 5,5-dithiobis (2-nitrobenzoic acid) [31]. A molar extinction coefficient (wavelength = 410 nm) of 13.6 × 10^3^ L^−1^·mol^−1^·cm^−1^ was used for the calculation of enzyme activity that was expressed in unit per liter. The intra-assay CV was 6.1% whereas the interassay CV was 9.8% [32].

Lp-PLA2 was assessed by using 2-thio PAF as substrate, which is hydrolyzed by the enzyme in sn-2 position, and the consequent formation of free thiols was detected by Ellman's procedure. A molar extinction coefficient (wavelength = 410 nm) of 13.6 × 10^3^ L^−1^·mol^−1^·cm^−1^ was used for the calculation of enzyme activity, expressed in unit per liter. The intra-assay CV was 4.8% whereas the interassay CV was 10.1% [32].

Serum levels of total cholesterol, triglycerides, HDL-C, LDL-C, and glucose were evaluated by routine laboratory methods.

### 2.3. Analysis of Lipoprotein Subclasses

Nine distinct lipoprotein subclasses were assessed in 25 *μ*L of serum by nondenaturated polyacrylamide gel electrophoresis and the Lipoprint system (Lipoprint LDL system and Lipoprint HDL system; Quantimetrix Corporation, Redondo Beach, CA) according to the manufacturer's specifications. The lipoprotein subclasses determined were as follows: very low-density lipoprotein (VLDL), three IDL subclasses (large, medium, and small IDL), two LDL subclasses (small and large), and three HDL subclasses (small, medium, and large). The gels were scanned, and the relative area for each band was measured and adjusted for total cholesterol level. In-depth description of this method is available here [33].

### 2.4. Statistical Analysis

Since the distribution of arylesterase, lactonase, and Lp-PLA2 analyzed by Kolmogorov-Smirnov test was skewed, the values were log transformed in order to approximate a normal distribution before being analyzed by parametric tests. Means of the variables examined were compared by using *t*-test while prevalence of categorical variables was compared by the *χ* ^2^ test. Pearson's correlation coefficient was used to evaluate the possible association between variables of interest. This test was followed by partial correlation or multiple regression analysis in order to check the independence of the observed univariate correlations. Multivariable logistic regression analysis was performed to evaluate whether arylesterase was independently related to T2DM. The covariates were included in the models if they were correlated with the dependent variable and/or not collinear with each other. In this test, arylesterase was classified as low if it was within the lower quartile. A *p* < 0.05 was considered statistically significant.

## 3. Results

### 3.1. PON1-Arylesterase, PON1-Lactonase, and Lp-PLA2 Activities in Controls and T2DM Patients (Whole Sample, *n* = 719)

The main demographic and clinical characteristics of the subjects enrolled in the present study are summarized in Table 1. Diabetics were significantly older and had higher BMI compared to controls; besides, this group presented higher prevalence of men, smokers, and subjects with hypertension.

**Figure 1:**
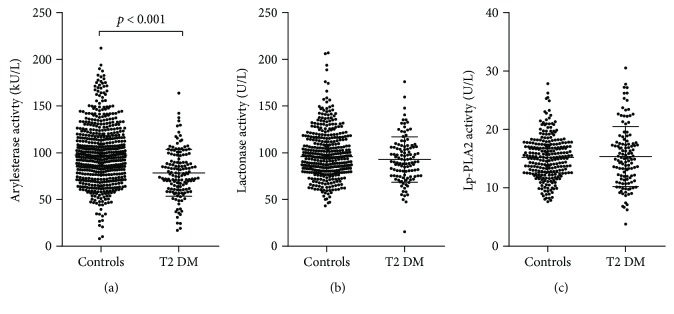
Scatter plots displaying PON1-arylesterase (a), PON1-lactonase (b), and Lp-PLA2 (c) activities in controls and T2DM patients.

Among the three serum enzyme activities assessed in this study, only PON1-arylesterase exhibited a significant change between groups. More specifically, as compared to controls, arylesterase was significantly (*p* < 0.001) decreased by 20% in participants with T2DM (78 ± 25 vs. 99 ± 31 kU/L) (Figure 1(a)). In contrast, either lactonase (*p* = 0.220) or Lp-PLA2 (*p* = 0.280) was not significantly different between the two groups (Figures 1(b) and 1(c)).

In order to check whether the observed decrease in arylesterase activity was influenced by confounding factors of PON1 such as age and gender, we compared this activity between subgroups of 85 controls and 85 diabetics with similar age and prevalence of women/men (Supplementary Table 1). From this analysis, it emerged that the difference in arylesterase was less marked than that found in the whole sample (11%), but still significant (*p* = 0.003).

### 3.2. Evaluation of the Possible Effect of Lipoprotein Subclasses on the Relationship between Arylesterase and T2DM (Lipoprint Subsample, *n* = 292)

The quantification of lipoprotein subfraction distribution was carried out on a subsample, Lipoprint, including 202 controls and 90 T2DM subjects, in order to (1) identify additional potential confounders of the association between arylesterase activity and T2DM and (2) explore the effect of T2DM on the distribution of PON1 activities and Lp-PLA_2_ on lipoprotein subclasses. Cases and controls included in this subset had similar demographic and clinical characteristics and equal difference pattern of the whole sample (Supplemental Table 2). Arylesterase, lactonase, and Lp-PLA_2_ activities also followed a similar trend, with only arylesterase showing a significant (*p* < 0.001) decrease in diabetics compared to controls (75 ± 20 vs. 96 ± 31 kU/L) (Supplementary Table 2).

With regard to lipid and lipoprotein subfraction profile, T2DM patients exhibited the typical atherogenic profile (Table 2). Indeed, they had higher levels of triglycerides and lower levels of HDL-C compared to the controls (*p* < 0.001), with the latter group presenting however an increase in total cholesterol and LDL (*p* < 0.001 vs. T2DM). Moreover, diabetics showed higher levels of proatherogenic small LDL-C (*p* < 0.001), lower levels of large LDL (*p* < 0.001), and lower LDL size (*p* < 0.01). Both large HDL-C and small HDL-C were decreased in diabetics (*p* < 0.001); regarding the relative percentages, large HDL-C decreased and small HDL-C increased in T2DM compared to controls (*p* < 0.001 for all). The other changes are as follows: (1) large IDL-C levels were higher in TD2M patients compared to controls while small IDL-C followed an opposite trend and (2) VLDL-C levels were higher in T2DM subjects (*p* < 0.001).

The above results along with the reports showing that PON1 reside in various lipoprotein subclasses [20, 23] prompted us to consider their relative serum concentration as possible confounders of the observed relationship between arylesterase and T2DM. To address this hypothesis, we first examined the association between this PON1 activity and lipoprotein subclasses in the whole population sample (Table 3). We found that serum levels of the enzyme activity were positively related with large HDL-C as expressed in concentration and percentage (*p* < 0.001 for both), medium and small HDL-C (*p* < 0.001 for both), large LDL-C (*p* < 0.05), mean LDL size (*p* < 0.001), and small IDL-C (*p* < 0.001); it was negatively related with the percentage of small HDL-C (*p* < 0.001) and that of small LDL-C (*p* < 0.05). Regarding the conventional lipid profile, arylesterase was associated positively with HDL-C (*p* < 0.001) and LDL-C (*p* < 0.05) and negatively with triglycerides (*p* < 0.001). Among the aforementioned correlations, only those involving large and medium HDL-C, LDL size, and small IDL-C remained significant after adjustment for age, gender, BMI, hypertension, and smoking (scatter plots of these associations were displayed in Supplementary Figure 1).

**Figure 2:**
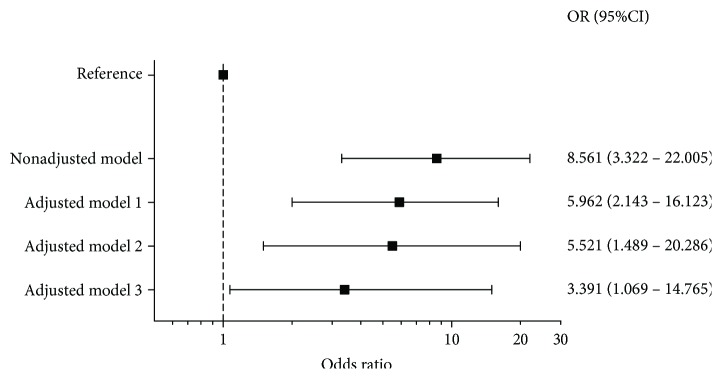
Box plots displaying unadjusted and multiadjusted odds ratio (95% confidence interval) for the association of a low PON1-arylesterase (activity level in the lower quartile) activity and T2DM model covariates: adjusted model 1: age, sex, smoking, body mass index, and hypertension; adjusted model 2: age, sex, smoking, body mass index, hypertension, and HDL-C; adjusted model 3: age, sex, smoking, body mass index, hypertension, large HDL-C, small HDL-C, LDL-particle size, and small IDL-C.

We next performed multivariable logistic regression to evaluate whether the independent correlates of arylesterase that emerged from the previous analysis could influence the association between this activity and T2DM (Figure 2). This analysis showed that low arylesterase (i.e., activity level in the lower quartile) confirmed the inverse association between this activity and T2DM that emerged from between-group comparison (Figure 1(a)). It also showed that while covariates such as age, gender, BMI, hypertension, smoking, and HDL-C did not markedly affect the association, the further inclusion of large HDL-C, medium HDL-C, LDL size, and small IDL-C led to a drastic decrease in the odds ratio (nonadjusted model, OR = 8.561, 95% CI 3.322–22.112; fully adjusted model, OR = 3.389, 95% CI 1.069–14.756).

### 3.3. Associations of Arylesterase, Lactonase, or Lp-PLA_2_ with Lipoprotein Subclasses in Controls and T2DM Patients (Lipoprint Subsample, *n* = 292)

To investigate the possible effect of T2DM on the distribution of PON1-arylesterase, PON1-lactonase and Lp-PLA2 across lipoprotein subclasses, we measured the correlation between these variables separately in both controls and T2DM subjects Table 4. For the sake of simplicity, this paragraph will only deal with those associations that remained significant after controlling for potential confounders (highlighted in bold in the table). Arylesterase was independently associated with HDL-C, large and medium HDL-C, mean LDL size, and small IDL-C (*p* < 0.05 after adjustment for all). The pattern changed among DM patients, where the correlations persisted for total cholesterol and HDL-C (*p* < 0.05 for both), small LDL-C (*p* < 0.05), all three IDL-C subclasses (*p* < 0.01 for all), and VDL-C (*p* < 0.01). Lactonase activity of PON1 was not correlated with any lipid/lipoprotein variables in controls but did correlate with mean LDL size (*p* < 0.05), large and medium IDL-C (*p* < 0.001 and *p* < 0.05, respectively), and VLDL-C (*p* < 0.01). Finally, Lp-PLA_2_ was weakly associated with total cholesterol and LDL-C (*p* < 0.05) in controls, with these two correlations becoming stronger (*p* < 0.01) in T2DM patients. Besides, within this group, the enzyme activity was also associated with small LDL-C (*p* < 0.01), mean LDL size (*p* < 0.05), large IDL-C (*p* < 0.01), and VLDL-C (*p* < 0.05).

## 4. Discussion

In overall agreement with previous reports [13, 14, 23], our study showed that PON1-arylesterase, but not PON1-lactonase or Lp-PLA2, activity was decreased in T2DM patients compared to controls. Of note, the observed difference between groups remained significant after adjustment for potential confounding factors, including lipoprotein subclasses. The subsequent analysis of the association between the enzyme activities and lipoprotein subclasses revealed that (1) Lp-pLA2 was associated with small LDL-C, large IDL-C, and VLDL-C in the T2DM group but not in controls and (2) arylesterase was associated only with large/medium HDL-C in controls and only with some of the more proatherogenic ApoB lipoproteins in T2DM subjects.

There is general consensus that the cholesterol component does not completely capture the vascular protective effect of HDL, which is indeed beyond its role in blood lipid transport [6, 34]. Other aspects of HDL functionality include the ability to contrast OxS and exacerbated inflammatory responses of immune cells involved in atherosclerotic processes [34–36]. PON1 has been widely shown to be one of the major contributors of HDL ability to contrast the oxidative challenges against their carrier, LDLs, macrophages, and endothelial cells [37, 38]. Lp-PLA2 is also reported to be associated with lipoproteins (in particular LDL) and to play a role in redox processes occurring in blood vessels [21, 39]. The hypothesized catalytic mechanism strictly recalls that of PON1; it can hydrolyze biomolecules resembling platelet-activating factors (PAF) (its natural substrate) such as phospholipids (PLPs) or containing oxidized fatty acyl groups [9]. Despite the abundant lines of experimental and clinical evidence on these two enzymes, there is still a high degree of vagueness regarding the various aspects of their biochemistry, physiological role, and impact on individual health [9, 40, 41].

One of the few certainties in this confusing landscape is the intimate interaction between the lipoprotein environment surrounding PON1 or Lp-PLA_2_ and their activities [9, 27, 42]; these two accessory proteins are anchored to lipids and some apolipoproteins which also coordinate and modulate their catalytic activity [43]. Recent improvements in separation techniques have highlighted that PON1 and Lp-PLA_2_ have a preferential, but not exclusive, distribution within HDL or LDL subclasses [20, 21, 23, 26, 44]. This may depend on the “broad” affinity of the enzymes with various apolipoproteins and lipid subspecies that may reach the most suitable combination in the small HDL_3_ in the case of PON1 and small LDL for Lp-PLA_2_. It was also demonstrated that when the composition and spatial location of phospholipid moiety or apolipoproteins such as APO A1 and A2 and Apo E change (as during HDL maturation), also the distribution of PON1 across HDL subclasses varies as well [21, 22, 39]. This “flux” of PON1 was observed to occur during HDL maturation, but it might be a phenomenon also associated with diseases typically featuring quantitative and qualitative abnormalities of lipid/apolipoprotein profiles, such as T2DM [12, 45]. In the present study, we focused on T2DM, not only because of the association with the aforementioned qualitative change in lipoproteins but also for the cumulating reports showing a disease-related alteration in PON1 and Lp-PLA_2_ activities [13, 21, 23].

To the best of our knowledge, this is the first study that evaluated whether the inverse association between arylesterase and T2DM is independent from such large spectrum of lipoprotein subclasses. Potential statistical interference of HDL subclasses on this association has been recently evaluated by Dullaart et al. [23] where the inverse relationship between PON1 and T2DM was modestly attenuated when the level of large HDL particles was included in the multivariable analysis. Our finding is overall consistent with the work of Dullaart et al., with a meaningful difference: the strength of the association largely decreased upon controlling for large and medium HDL-C and small IDL-C (from OR = 5.522, 95% CI 1.489–20.426 to OR = 3.389, 95% CI 1.069–14.756), suggesting that the distribution of PON1 in lipoprotein subclasses can, in part, explain the observed relationship. In addition, the data regarding arylesterase and lactonase were highly discrepant but this outcome was not either surprising or unprecedented [36]. Arylesterase, although referred as one of the two promiscuous activities of PON1 (the other is paraoxonase), is more frequently measured in epidemiological studies compared to the putative physiological activity, lactonase [28, 46, 47]. Arylesterase is minimally influenced by some prevalent PON1 genetic polymorphisms, discloses low interindividual variability, and is regarded as a better surrogate of PON1 concentration than the other two activities [47]. Furthermore, regarding the lack of significant association between Lp-PLA2 and T2DM, data on the association with T2DM and related CV complications are highly variable and divergent [16, 17, 21], and besides, it is still not clear whether high/low levels of Lp-PLA2 are beneficial or detrimental for human health [32, 48]. Some authors suggest that the possible explanation of this recurrent paradox may be related to the distribution of Lp-PLA2 among lipoprotein classes and subclasses [49]. In particular, Lp-PLA2 might be anti-inflammatory when is complexed with HDL, whereas it is proinflammatory (the hydrolysis of oxidized lipids generates the cytotoxic lysophosphatidylcholine [9]) when it resides in ApoB-containing lipoproteins [9, 32, 50].

In order to address the possibility of influence of the diabetic state over the distribution of Lp-PLA2 and PON1 activities across lipoprotein subspecies, we assessed the simple and adjusted correlation coefficients separately in controls and T2DM subjects. The finding that Lp-PLA2 was associated to the small LDL-C, one of the most important risk factors of CVD, is consistent with some studies on isolated lipoproteins showing that in healthy people [51, 52] and, mostly, in diabetics [21], the enzyme is preferentially located in this LDL subclass. Failure in detecting a significant correlation of Lp-PLA2 with small LDL-C in controls may be the result of the low levels of this subclass in this group (Table 2). Alternatively, it can also be hypothesized that ApoB lipoproteins may be enriched in active Lp-PLA2 in T2DM patients and this could reflect in a further selective increase in proatherosclerotic potentials of these particles. In turn, this change might contribute to the excess risk for CVD in people with diabetes.

Regarding PON1, the disappearance and appearance of association with large/medium HDL-C and the more proatherogenic ApoB lipoproteins, respectively, in T2DM csubjects may have two, not necessarily antithetic, explanations.

First, the phenomenon could merely depend on the change in lipoprotein profile. Within this assumption, the lack of correlation between arylesterase and large/medium HDL-C in the T2DM group may be the result of the marked decrease in the concentration of these subclasses (Table 2) and, thus, of the fraction of PON1 complexed with these particles. On the other hand, the exclusive association of arylesterase and lactonase with large/medium IDL, small LDL, and VLDL among T2DM patients might simply reflect the concomitant increase in the levels of these lipoproteins. As a proof of concept, PON1 has been detected within VLDL, VLDL remnants (i.e., large IDL), and small LDL in healthy individuals [19], but not in a sufficient amount to significantly contribute in arylesterase/lactonase total serum activities.

It can be also speculated that the redistribution of PON1 among HDL subclasses is caused by the disease itself. In T2DM patients, PON1 is still bound in HDL, as suggested by the strong correlation between arylesterase and total HDL-C, but most likely, it is more evenly distributed among the subspecies of this lipoprotein compared to controls. Besides, it has been reported that glycation and oxidation of HDL or directly of PON1 occurring in T2DM may cause the detachment of the enzyme from its host and, as consequence, affect its activity [53].

Some important limitations of the study need to be underpinned. Firstly, the procedure for assessing the distribution of lipoprotein measures the cholesterol amount associated to each subfraction subclasses, but not the particle number. Notwithstanding this limitation, Quantimetrix Lipoprint has been consistently described to afford a reliable quantitative determination of LDL/HDL/IDL subfractions [33, 54]; as proof of concept, the observed distribution of lipoprotein subclasses in our population sample is comparable with that reported by a number of studies including those dealing with particle quantification. Secondly, we were not able to measure the real fraction of PON1 or Lp-PLA2 activity in each lipoprotein subclass. However, with some exceptions [6, 20], our results on nondiabetic subjects are in overall concordance with other studies dealing with the detection of enzyme activity/mass in isolated subfractions [12, 19, 23, 55]. Thirdly, we cannot exclude that other confounders besides those considered in the multivariable analyses could bias our results. This is the case of putative modulators of PON1 activity such as hormonal therapies (e.g., oral contraceptives), nutrient components (in particular, vitamins E and C), drugs (e.g., statins) or still not precisely identified environmental pollutants. Thirdly, the cross-sectional design did not allow to define a cause-effect relationship between enzyme activities, lipoprotein subclasses, and TD2M.

## 5. Conclusion

In conclusion, the present community-based population study showed that PON1-arylesterase activity is inversely associated with T2DM. Notably, we found that this relationship was independent of several confounding factors, including the lipoprotein subclasses that may carry PON1 itself. Our study also showed that the occurrence of T2DM could coincide with a shift of PON1 and Lp-PLA2 towards the more proatherogenic lipoprotein subclasses. The existence of a possible cause-effect link between decreased PON1 activities and its redistribution across lipoprotein subclasses required further investigations.

## Figures and Tables

**Table 1:** Main characteristics of controls and T2DM subjects (total sample, *n* = 719).

	T2DM (*n* = 145)	Controls (*n* = 574)	*p* value
Age (years)	69 ± 11	64 ± 13	<0.001
Gender (women/men)	85/60	419/155	<0.001
BMI (kg/m^2^)	33 ± 6	25 ± 5	<0.001
Smoking (% never/ex/current)	39/44/17	66/13/21	<0.001
Glucose (mg/dL)	147 ± 36	96 ± 10	<0.001
Hypertension (%)	85	40	<0.001

Data are expressed as means ± standard deviations for continuous variables and number or percentage within the group for categorical variables. BMI: body mass index.

**Table 2:** Lipid profile and distribution of lipoprotein subfractions in controls and T2DM subjects included in lipoprotein subsample.

	T2DM (*n* = 90)	Controls (*n* = 202)	*p* value
Total cholesterol (mg/dL)	188 ± 47	208 ± 32	<0.001
LDL-C (mg/dL)	114 ± 43	138 ± 30	<0.001
HDL-C (mg/dL)	34 ± 10	52 ± 9	<0.001
Triglycerides (mg/dL)	206 ± 91	88 ± 41	<0.001
*HDL subfractions*			
Large HDL-C (%)	25 ± 7	33 ± 7	<0.001
Medium HDL-C (%)	47 ± 3	45 ± 4	0.001
Small HDL-C (%)	28 ± 6	21 ± 5	<0.001
Large HDL-C (mg/dL)	9 ± 4	18 ± 6	<0.001
Medium HDL-C (mg/dL)	16 ± 5	23 ± 4	<0.001
Small HDL-C (mg/dL)	9 ± 3	11 ± 3	<0.001
*LDL subfractions*			
Large LDL-C (mg/dL)	51 ± 19	66 ± 20	<0.001
Small LDL-C (mg/dL)	17 ± 12	10 ± 10	<0.001
Mean LDL-C particle size (Å)	262 ± 5	268 ± 4	<0.01
*IDL subfractions*			
Large IDL-C (mg/dL)	14 ± 5	12 ± 4	<0.001
Medium IDL-C (mg/dL)	15 ± 7	15 ± 6	0.200
Small IDL-C (mg/dL)	12 ± 6	17 ± 5	<0.001
VLDL-C (mg/dL)	45 ± 13	34 ± 8	<0.001

Data are expressed as means ± standard deviations. HDL-C: high-density lipoprotein cholesterol; LDL-C: low-density lipoprotein cholesterol; VLDL-C: very low-density lipoprotein cholesterol; IDL-C: intermediate-density lipoprotein cholesterol.

**Table 3:** Simple (*r*) and partial (*r*_partial_) Pearson's correlation coefficients of the relationship between PON1-arylesterase and serum lipids and lipoprotein subfractions.

	Pearson's correlation coefficient (*r*)	Partial Pearson's correlation coefficient^#^ (*r*_partial_)
Total cholesterol	0.187	0.074
*HDL-C*	0.394^∗∗^	*0.239* ^∗∗^
LDL-C	0.194^∗^	0.063
Triglycerides	−0.275^∗∗^	−0.100
Large HDL-C (%)	0.268^∗∗^	0.077
Medium HDL-C (%)	−0.141	0.014
Small HDL-C (%)	−0.254^∗∗^	−0.111
*Large HDL-C (mg/dL)*	0.381^∗∗^	*0.193* ^∗^
*Medium HDL-C (mg/dL)*	0.376^∗∗^	*0.234* ^∗∗^
Small HDL-C (mg/dL)	0.129^∗^	0.065
Large LDL-C	0.209^∗^	0.050
Small LDL-C	−0.175^∗^	0.092
*Mean LDL particle size*	0.295^∗∗^	*0.182* ^∗^
Large IDL-C	0.071	0.053
Medium IDL-C	0.114	0.110
*Small IDL-C*	0.375^∗∗^	*0.268* ^∗∗^
VLDL-C	−0.061	0.050

^∗^ *p* < 0.05; ^∗∗^ *p* < 0.001; significant partial correlation coefficients are highlighted in italics; ^#^covariates: age, sex, smoking, hypertension, and BMI. HDL-C: high-density lipoprotein cholesterol; LDL-C: low-density lipoprotein cholesterol; VLDL; very low-density lipoprotein cholesterol; IDL-C, intermediate-density lipoprotein cholesterol.

**Table 4:** Pearson's correlation coefficients of the relationship of arylesterase, lactonase, or Lp-PLA2 with serum lipids or lipoprotein subfractions in controls and in type 2 DM subjects.

Lipoproteins	Controls			Type 2 diabetes		
	Arylesterase *r*	Lactonase *r*	Lp-PLA2 *r*	Arylesterase *r*	Lactonase *r*	Lp-PLA2 *r*
Total cholesterol	0.172^∗^	0.201^∗^	*0.265* ^∗∗^	*0.315* ^∗^	0.302^∗∗^	*0.411* ^∗^
HDL-C	*0.404* ^∗∗^	0.159^∗^	−0.100	*0.464* ^∗∗^	0.168	−0.060
LDL-C	0.174^∗^	0.154^∗^	*0.232* ^∗∗^	0.063	0.233^∗^	*0.388* ^∗∗^
Triglycerides	−0.169^∗^	−0.009	0.183^∗^	−0.246^∗^	0.212^∗^	0.189
Large HDL-C (%)	0.192^∗^	0.011	0.108	−0.046	−0.064	−0.141
Medium HDL-C (%)	−0.094	−0.019	−0.096	0.013	−0.024	−0.021
Small HDL-C (%)	−0.195^∗^	0.002	−0.045	0.073	0.100	0.151
Large HDL-C (mg/dL)	*0.257* ^∗^	−0.068	0.077	0.124	0.067	−0.134
Medium HDL-C (mg/dL)	*0.223* ^∗^	0.128	−0.011	0.245^∗^	0.187	−0.124
Small HDL-C (mg/dL)	−0.163	0.108	0.007	0.226^∗^	0.215^∗^	−0.046
Large LDL-C	−0.041	0.146	0.312^∗∗^	0.143	0.113	0.104
Small LDL-C	−0.196^∗^	−0.044	0.117	*0.223* ^∗^	*0.263* ^∗^	*0.314* ^∗∗^
Mean LDL particle size	*0.244* ^∗^	0.074	−0.059	0.079	−0.191	**−** *0.280* ^∗∗^
Large IDL-C	0.098	0.097	0.051	*0.285* ^∗^	*0.400* ^∗∗^	*0.314* ^∗^
Medium IDL-C	0.037	0.097	−0.004	*0.367* ^∗∗^	*0.314* ^∗^	0.205
Small IDL-C	*0.274* ^∗∗^	0.188^∗^	−0.018	*0.309* ^∗^	0.077	0.056
VLDL-C	−0.008	0.005	−0.086	*0.288* ^∗∗^	*0.338* ^∗∗^	*0.283* ^∗∗^

^∗^ *p* < 0.05; ^∗∗^ *p* < 0.001; significant partial correlation coefficients (covariates: age, sex, smoking, hypertension, and BMI) are highlighted in italics. HDL-C: high-density lipoprotein cholesterol; LDL-C: low-density lipoprotein cholesterol; VLDL: very low-density lipoprotein cholesterol; IDL-C: intermediate-density lipoprotein cholesterol. Lactonase was measured in 152/202 controls; Lp-PLA2 was measured in 111/202 controls.

## Data Availability

The data (included in an excel database) used to support the findings of this study are available from the corresponding author upon request.

## Supplementary Materials

Supplementary Materials Supplementary Figure 1: (figure legend) scatter plots displaying the correlation between Log10 PON1-arylesterase activity and HDL-C (A), large HDL-C (B), medium HDL-C (C), mean LDL particle size (D), and small IDL-C (E). Supplementary Table 1: main characteristics and serum arylesterase and lactonase activities of PON1, in controls and type 2 DM subjects with similar age and gender distribution. Supplementary Table 2: main characteristics and serum levels of PON1-arylesterase, PON1-lactonase, and Lp-PLA2 activities of controls and T2DM subjects (Lipoprint subsample, *n* = 292).
