# Supplementary material for: Distribution of Paraoxonase-1 (PON-1) and Lipoprotein Phospholipase A2 (Lp-PLA2) across Lipoprotein Subclasses in Subjects with Type 2 Diabetes
Source: Oxid Med Cell Longev. 2018 Nov 5;2018:1752940. doi: 10.1155/2018/1752940 (PMC6247389; doi:10.1155/2018/1752940)
Supplement: Supplementary Materials — Supplementary Figure 1: (figure legend) scatter plots displaying the correlation between Log10 PON1-arylesterase activity and HDL-C (A), large HDL-C (B), medium HDL-C (C), mean LDL particle size (D), and small IDL-C (E). Supplementary Table 1: main characteristics and serum arylesterase and lactonase activities of PON1, in controls and type 2 DM subjects with similar age and gender distribution. Supplementary Table 2: main characteristics and serum levels of PON1-arylesterase, PON1-lactonase, and Lp-PLA2 activities of controls and T2DM subjects (Lipoprint subsample, n = 292). [file 1752940.f1.pdf]

Supplementary Figure 1.

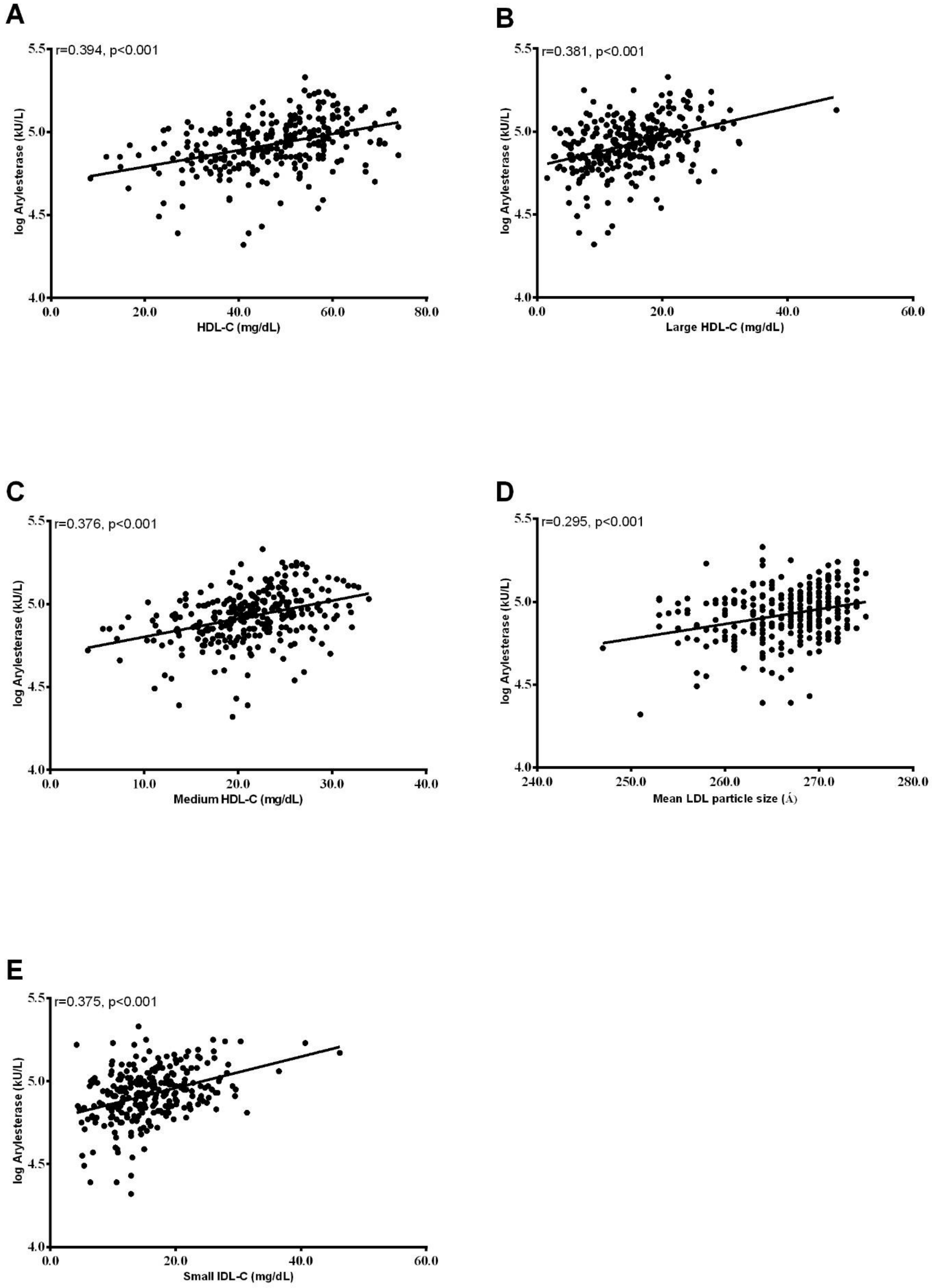

Supplemental Table 1. PON1-Arylesterase in Controls and T2DM subjects with similar age and gender prevalence

	T2DM* (n=85)	Control* (n= 85)	p value
Arylesterase (KU/L)	73 ± 24	82 ± 20	0.003
Age (years)	63 ± 8	65 ± 9	0.700
Gender (women/men)	35/50	35/50	1.00
BMI (Kg/m^2)	31 ± 4	27 ± 5	<0.001
Smoking (% never/ex/current)	39 /43/18	35/37/18	<0.001
Glucose (mg/dL)	145 ± 35	99 ± 9	<0.001
Hypertension (%)	80	36	<0.001

Data are expressed as means ± standard deviations for continuous variables and number or percentage within the group for categorical variables. Abbreviations: BMI, body mass index

*Subsets extracted from total sample

Supplemental Table 2. Main characteristics and serum levels of PON1-Arylesterase, PON1-Lactonase and Lp-PLA2 activities of Controls and T2DM subjects (Lipoprint subsample, n=292)

	T2DM (n=90)	Controls (n= 202)	p value
Age (years)	66 ± 10	56 ± 11	<0.001
Gender (Women/Men)	35/55	139/63	<0.001
BMI (Kg/m^2)	32 ± 5	26 ± 5	<0.001
Glucose (mg/dL)	149 ± 40	98 ± 12	<0.001
Smoking (% never/ex/current)	34 /46	49/1/49	<0.001
Hypertension (%)	93	58	<0.001
Dyslipidemia (%)	98	54	<0.001
Enzyme activities			
Arylesterase (KU/L)	75 ± 20	96 ± 31	<0.001
Lactonase (U/L)[#]	94 ± 25	98 ± 22	0.274
Lp-PLA2 (U/L)[#]	15 ± 5	14 ± 3	0.431

Data are expressed as means ± standard deviations for continuous variables and number or percentage within the group for categorical variables

Abbreviations: BMI, body mass index

[#]Lactonase was measured in 152/202 Controls; Lp-PLA$_2$ was measured in 111/202 Controls
